# Unlocking the potential of tumor-targeting peptides in precision oncology

**DOI:** 10.32604/or.2025.062197

**Published:** 2025-06-26

**Authors:** HAFIZ MUHAMMAD REHMAN, SIDRA AHMAD, AZEEM SARWAR, HAMID BASHIR

**Affiliations:** 1 University Institute of Medical Lab Technology, Faculty of Allied Health Sciences, The University of Lahore, Lahore, 54590, Pakistan; 2 Centre for Applied Molecular Biology, 87-West Canal, Bank Road, University of the Punjab, Lahore, 53700, Pakistan

**Keywords:** Targeting peptides, Peptide therapeutics, Anti-cancer peptide sources, Cancer cell mechanism, Cancer informatics

## Abstract

Targeted cancer therapy has emerged as a promising alternative to conventional chemotherapy, which is often plagued by poor selectivity, off-target effects, and drug resistance. Among the various targeting agents in development, peptides stand out for their unique advantages, including minimal immunogenicity, high tissue penetration, and ease of modification. Their small size, specificity, and flexibility allow them to target cancer cells while minimizing damage to healthy tissue selectively. Peptide-based therapies have shown great potential in enhancing the efficacy of drug delivery, improving tumor imaging, and reducing adverse effects. With cancer responsible for millions of deaths worldwide, the development of peptide-based therapeutics offers new hope in addressing the limitations of current treatments. As detailed studies on different aspects of targeting peptides are crucial for optimizing drug development, this review provides a comprehensive overview of the literature on tumor-targeting peptides, including their structure, sources, modes of action, and their application in cancer therapy—both as standalone agents and in fusion drugs. Additionally, various computational tools for peptide-based tumor-targeting drug design and validation are explored. The promising results from these studies highlight peptides as ideal candidates for targeted cancer therapies, offering valuable insights for researchers and accelerating the discovery of novel anti-tumor peptide base drug candidates.

## Introduction

Cancer remains one of the leading causes of death worldwide, with approximately 20 million new cases and over 9.7 million deaths till 2022 [1]. Despite advancements in early detection and therapeutic interventions, cancer treatment continues to be a significant challenge for clinicians and researchers. Traditional therapies such as chemotherapy, radiation therapy, and surgery are often limited by their lack of selectivity toward tumor cells, leading to severe side effects and damage to healthy tissue [2]. Chemotherapeutic agents, while effective at targeting rapidly dividing cells, often result in off-target toxicity, as they do not discriminate between cancerous and healthy tissues. This lack of specificity frequently leads to adverse effects, including hair loss, nausea, and immune suppression [3]. Furthermore, many cancer cells develop resistance to chemotherapy over time through mechanisms such as drug efflux, gene mutations [4], or alterations in drug metabolism [5]. Targeted cancer therapies have emerged as a promising solution to these challenges. Unlike traditional chemotherapeutics, which non-selectively attack rapidly dividing cells, targeted therapies are designed to interact with specific molecules involved in the growth and survival of cancer cells, sparing healthy tissues and reducing systemic toxicity [6]. These therapies often exploit unique molecular signatures—such as overexpressed receptors, mutated proteins, or altered signaling pathways—that distinguish cancer cells from their normal cells [7]. These therapies offer the potential for improved efficacy and reduced toxicity by selectively attacking cancer cells while minimizing damage to healthy tissues. However, the development of such therapies requires a deep understanding of the molecular mechanisms underlying tumorigenesis and the identification of tumor-specific markers. In recent years, peptides have emerged as highly promising candidates for targeted cancer therapy. These small, versatile molecules can be engineered to selectively bind to receptors that are overexpressed on the surface of tumor cells [8]. Due to their small size, low immunogenicity, and exceptional tissue penetrability, peptides are particularly attractive for drug delivery systems [9]. One of the earliest successes in targeted cancer treatment was imatinib, a tyrosine kinase inhibitor that targets the breakpoint cluster region and Abelson murine leukemia (BCR-ABL) fusion protein in chronic myeloid leukemia (CML) [10]. This shift toward targeted therapies, including peptide-based strategies, reflects an increasing understanding of cancer as a complex and heterogeneous disease. By exploiting the molecular differences between cancerous and normal tissues, targeted therapies offer the potential for more effective, less toxic treatments. This approach is central to the concept of precision medicine, where therapies are tailored to the specific molecular profile of each patient’s tumor, paving the way for more personalized and impactful cancer care.

Monoclonal antibodies are engineered to mimic the immune system’s ability to target specific antigens on cancer cells. They have become a keystone of precision oncology due to their ability to block signaling pathways, induce immune-mediated cytotoxicity, or deliver cytotoxic payloads directly to tumor cells [11]. For example, trastuzumab, a mAb targeting human epidermal growth factor receptor 2 (HER2), has significantly improved outcomes in HER2-positive breast cancer [12]. Recent advancements include the development of bispecific antibodies, which can simultaneously engage two different antigens, enhancing their therapeutic efficacy [13]. Monoclonal antibody fragments, such as Fab (antigen-binding fragment) and scFv (single-chain variable fragment), offer advantages over full-length mAbs, including smaller size, better tissue penetration, and reduced immunogenicity [14]. These fragments are particularly useful in targeting solid tumors and have been employed in imaging and drug delivery systems [15]. For instance, nanobodies, derived from camelid heavy-chain antibodies, have shown promise in targeting hard-to-reach tumor sites due to their small size and high stability [16].

Gene therapy involves the introduction, removal, or alteration of genetic material within a patient’s cells to treat disease. In oncology, this approach can be used to replace mutated genes that cause cancer with healthy copies, inactivate genes that promote cancer growth, or introduce new genes to help the body fight cancer. Recent developments have focused on enhancing delivery methods and ensuring the precise integration of therapeutic genes to maximize efficacy and safety [17]. Cell therapy, particularly chimeric antigen receptor T-cell therapy, has revolutionized cancer treatment by modifying a patient’s T cells to express receptors specific to cancer antigens. These engineered T cells are then expanded and reintroduced into the patient to target and destroy cancer cells. Advancements in this field aim to improve the efficacy of chimeric antigen receptor T-cell therapies against solid tumors and reduce associated toxicities [18].

In this review, we aim to provide a detailed analysis of the latest research on tumor-targeting peptides, including their structural features, sources, and modes of action. We also explore the current and potential applications of peptides in cancer therapy, both as standalone treatments and in combination with small-molecule drugs or other therapeutic agents. Furthermore, we discuss the computational tools and techniques used to design peptide-based cancer therapies, offering examples of how these tools have been successfully implemented in drug development. This study will facilitate the discovery of novel peptides and improve the design of effective, targeted cancer treatments, addressing the ongoing challenges in oncology.

## Structure of Targeting Peptides

Peptide bonds bind short chains of amino acids together to form peptides. Their stability and biological function are greatly influenced by their structural characteristics, including length, amino acid sequence, and total charge [19]. The peptide’s general characteristics and interactions with target receptors are determined by its fundamental structure, or the arrangement of its amino acids [20]. This sequence can be altered to improve binding specificity and affinity [21]. Alpha-helices and beta-sheets are examples of secondary structures that affect peptide stability and conformational flexibility, both of which are critical for efficient interaction with tumor targets [22].

α-Helical anticancer peptides (ACPs), such as Magainin II from the African clawed frog [23], are extensively studied for their potent anti-tumor activity at micromolar concentrations, targeting negatively charged tumor cell membranes through electrostatic and hydrophobic interactions. These peptides, with a high net positive charge (+2 to +9 C) due to lysine and arginine residues, disrupt tumor cell membranes and induce necrosis [24]. Magainin II has demonstrated efficacy against bladder cancer and lung cancer cells (IC50 110 µg/mL) while being non-toxic to normal epidermal cells [25]. Other α-helical peptides, such as A12L/A20L and Aurein, isolated from frog secretions, also exhibit significant specificity and anti-tumor activity against various cancers, including glioblastoma [26]. The hydrophobic arc dimensions of these peptides are crucial for their selectivity, enabling them to degrade the plasma and mitochondrial membranes of tumor cells while sparing non-cancerous cells. β-pleated sheet ACPs, primarily derived from plants and animals, are structurally stable due to the presence of two or more disulfide bonds. Although less prevalent than α-helical peptides, they exhibit significant anti-tumor activity with minimal toxicity to normal tissues. Peptides like SVS-1 adopt a β-pleated conformation upon contact with tumor cell surfaces, forming pores and disrupting membranes, with activity against cell lines such as A549 (human lung epithelial cell line), KB (subline of the HeLa cervical cancer), MCF-7 (human breast cancer cell), and MDA-MB-436 (MD Anderson-Metastatic Breast-436 cell line) [27]. LfcinB, derived from cattle lactoferrin [28], and HNP-1, targeting PC-3 prostate cancer cells (IC50 2.2 µM), exemplify the potential of β-pleated ACPs. Despite being less potent than α-helical peptides, their lower toxicity makes them promising candidates for anti-cancer drug development. Random coil ACPs, rich in proline and glycine, lack a defined secondary structure and exhibit moderate anti-tumor activity compared to β-pleated sheet peptides. Examples include PR-39, isolated from neutrophils, which shows antiproliferative effects on tumor cells but also affects normal kidney cells, and its mutant PR-35, which reduces cytotoxicity while maintaining biological activity [29]. Peptides like Pep27anal2, with random coil conformations, penetrate cell membranes and induce apoptosis independently of caspase and cytochrome c [30]. Insect-derived random coil peptides display significant therapeutic potential, and Alloferon has demonstrated antiviral and immunomodulatory effects, enhancing NK cell activity and interferon synthesis. Despite their lower cytotoxicity on healthy tissues compared to α-helical and β-pleated ACPs, random coil peptides hold promise for therapeutic applications. Tertiary structure, the three-dimensional folding of the peptide, is critical for precise receptor binding and cellular uptake [31]. By strategically modifying these structural elements, researchers can improve peptide stability, enhance target selectivity, and optimize therapeutic efficacy, making peptides more effective in specifically targeting and treating cancer cells.

### Anti-cancer peptide discovery

High-throughput screening (HTS) in cancer peptide discovery is a technology-driven approach used to rapidly identify peptides with potential therapeutic or diagnostic applications in oncology.

### Phage display technology

Phage display technology has significantly advanced in recent years, enhancing its application in anticancer peptide discovery [32]. This technique involves engineering bacteriophages to display peptides or proteins on their surface, facilitating the screening of extensive libraries to identify sequences that specifically interact with cancer-related targets [33]. Through a process called biopanning, peptides that bind to immobilized cancer targets—such as receptors, enzymes, or tumor biomarkers—are enriched via iterative rounds of binding, washing, elution, and amplification [34]. Sequencing the phage DNA reveals the peptide sequences, which are subsequently synthesized and evaluated for therapeutic or diagnostic potential [35]. For instance, phage display has been instrumental in identifying tumor-homing peptides that enhance targeted drug delivery and imaging [36]. Despite challenges such as *in vitro* bias, target accessibility issues, and the need for peptide optimization [37], phage display continues to be a powerful tool in oncology research, contributing to the discovery of novel peptides for cancer therapy and diagnosis.

### Yeast display and combinatorial peptide library

Yeast display technology facilitates the identification of peptides with high specificity and affinity for cancer-related targets. It has been widely used in anticancer peptide discovery due to its robustness, ability to express complex peptides, and compatibility with fluorescence-activated cell sorting (FACS) for precise selection [38]. The system typically involves the fusion of a peptide library to a scaffold protein, such as the Aga2p subunit of the Aga1p–Aga2p adhesion complex in *Saccharomyces cerevisiae* [39]. This allows peptides to be displayed on the yeast surface while maintaining proper folding and functionality. The displayed peptides are screened against cancer-related targets, such as membrane receptors or tumor-specific antigens, using iterative rounds of selection (biopanning). High-affinity binders are enriched through FACS or magnetic bead-based separation [40]. Unlike phage display, the cost-effectiveness and reproduciblele yeast cells provide post-translational modifications and proper folding, quantitative Screening, library diversity and affinity maturation which enables the generation of large peptide libraries (~10^9^ variants) for iterative selection to improve affinity and specificity [41]. Previously, yeast display technology had identified peptides that inhibit the PD-1/PD-L1 interaction, thereby enhancing T-cell activation against tumors. For instance, researchers have engineered high-affinity variants of the PD-1 ectodomain using yeast surface display. These engineered proteins exhibit superior tumor penetration and efficacy compared to traditional anti–PD-L1 antibodies [42]. However, there are limitations such as size constraints, being more suitable for short peptides and small proteins, and lower display efficiency compared to phage display. Additionally, it may struggle with displaying non-natural peptides containing unnatural amino acids or post-translational modifications [43]. Some other HTS includes peptide nucleic acid (PNA)-encoded solution phase peptide library which offers cost-effective lead ligand optimization and multiple-use potential but is limited by its complex DNA decoding process and non-commercial availability [44]. Peptide microarrays provide cost-effective screening with replicable peptide chips but are restricted in library size and not reusable for subsequent assays [45]. Bacteria-display allows quantitative screening using fluorescence-activated cell sorting (FACS) without requiring reinfection but may suffer from surface interference during peptide binding [46]. Ribosome- or mRNA-display systems enable high library diversity and easy mutagenesis but have low display efficiency [47]. Chemical libraries, such as the one-bead-one-compound approach, facilitate efficient peptide synthesis and screening beyond natural amino acids, but their linker effects remain unpredictable, and they are not suitable for *in vivo* selection [48]. These methods collectively enhance peptide discovery for biomedical applications, particularly in anticancer research.

## Sources of Targeting Peptides

### Natural peptide sources

Natural peptides derived from various sources have shown significant potential in targeting cancer cells due to their specificity, binding affinity, and therapeutic efficacy. These peptides can be isolated from diverse sources such as animals, plants, microorganisms, and marine organisms, each offering unique properties that can be harnessed for cancer treatment.

### Animal-derived peptides

The peptides listed in Table 1 demonstrate diverse anti-cancer activities by targeting specific pathways, signalling molecules, or cellular processes across various tumor types. Pardaxin, derived from the Red Sea flatfish, inhibits nuclear factor kappa-light-chain-enhancer of activated B cells (NF-κB) signaling in murine fibrosarcoma cells, showing significant anti-tumor effects both *in vitro* and *in vivo* [49]. AUNP-12, a PD-1 antagonist, enhances immune cell functions and is effective against melanoma, breast, and kidney cancers [50]. Dermaseptin-PD-2, sourced from the Phyllomedusine leaf frog, impedes proliferation in glioblastoma cells [51], while XLAsp-P1 from the South African clawed frog exhibits inhibitory activity against breast cancer cells [52]. Melittin, a peptide from honeybee venom, exhibits potent anticancer activity by causing membrane disruption and promoting cell death in various cancer types [53]. Pentadactylin, identified in the pepper frog, demonstrates cytotoxic effects on murine melanoma cells, though with limited specificity [54]. Marine-derived peptides, such as the oyster peptide and TFD glycoprotein from Pacific cod, induce DNA damage and apoptosis in colon and prostate cancer cells, respectively [55]. Blood clam-derived peptides, along with those from skate, freshwater crocodile [56], and *Sinonovacula constricta*, show potent cytotoxic and apoptotic activities across a range of cell lines, including cervical, lung, and prostate cancers [57]. Notably, lactoferrin fragments regulate cell death in leukemia and breast cancer cells [58], while A5-1 peptide inhibits integrin-mediated tumor neovascularization [59]. Additionally, Diazonamide A from an unspecified animal source inhibits tubulin polymerization, showing efficacy in breast cancer cells [60]. Collectively, these peptides represent promising candidates for targeted cancer therapies, leveraging mechanisms such as apoptosis, DNA damage, cytotoxicity, and immune modulation.

**Table 1:** List of selected animal derived anti-cancer peptides

Peptide name	Primary sequence	Target	Tumor	Mechanism	Animal source	References
Pardaxin	GFFALIPKIISSPLFKTLLSAVGSALSSSGGQE	NF-κB signalling	Murine fibrosarcoma	Evaluation of anti tumor effects on MN-11 both *in vitro* and *in vivo*	Red sea flatfish	[49]
AUNP-12	SNTSESFKFRVTQLAPKAQIKE	PD-1	Melanoma, Breast, and Kidney cancers	Disrupting the PD-1-PD-L1/2 interaction to enhance immune cell proliferation and effector functions.	Animal	[50]
Glycoprotein	TFD (Galβ1, 3GalNAc)	PC3	Prostate cancer	Apoptosis	Pacific cod	[55]
	PSLVGAPPVGKLTL	HT-29 cell line	Human colon carcinoma	DNA damage, morphological alterations, cell proliferation inhibition	Oyster	[69]
Dermaseptin-PD-2	GMWSKIKNAGKAAAKAAAKAAGKAALDAVSEAI	U251MG cell line	Human neuronal glioblastoma	Impeded the proliferation of H157, U251MG, and PC-3 cells	Phyllomedusine leaf frog	[51]
XLAsp-P1	DEDDD	_	Breast cancer cells	Activity inhibiting breast cancer cells	South African clawed frog	[52]
Pentadactylin	GLLDTLKGAAKNVVGSLASKVMEKL	B16F10 cell line	Murine melanoma	B16F10 cells exposed to cytotoxic activity without high specificity	Pepper frog	[54]
Lactoferrin	LFcinB-CLICK	Jurkat and MDA-MB-231	T-leukemia and breast	Regulated cell death	Bovine serum	[58]
KT2	NGVQPKYRWWRWWRRWW	HeLa and CaSki cell lines	Cervix cancer	Apoptosis-induced cell death	Fresh water crocodile	[56]
Diazonamide A	_	MCF-7	Breast cancer cells	Potent cytotoxic activity and inhibits tubulin polymerization	Animal	[60]
EYGF-33	YPSPV	Caco-2	Colon cancer	Programmed cell death	Egg yolk	[70]
ACFP	_	SKOV3	Ovarian cancer	Controlled cell death	Bovine milk protein	[71]
A5-1	VILVLF	Integrin α5β1	Tumoral neovessels	Inhibits integrin-fibronectin interaction and suppresses endothelial cell functions	Animal	[72]
SCH-P9 and SCH-P10	Leu-Pro-Gly-Pro and Asp-Tyr-Val-Pro	DU-145 and PC-3	Prostate cancer	Apoptosis	*Sinonovacula constricta*	[73]
Gelatin	BCH-P1	B16F10	Murine hepatoma	Self-destruction of cells	Bovine skin collagen	[74]
KT2	NGVQP-KYRWWRW WRRWW	HCT116	Colon	Cellular suicide	Crocodile	[75]
TFD100	TPGAYRQWQKEV	Galectin-3 (Gal3)	Prostate cancer cells	Prevented angiogenesis, PC3 adherence to endothelial cells, and T-cell apoptosis	Fish	[55]

### Plant-derived peptides

Plant-derived peptides often exhibit potent anticancer activities by targeting key molecular pathways and inducing specific cellular mechanisms. NDF, isolated from the common bean suppresses colon cancer cell proliferation by modulating proteins involved in cell cycle regulation, such as p53 and p21, and promoting apoptosis through pathways involving BAD, cytC, and caspase-3 [61]. Chickpea-derived CPe-III elevates p53 levels, effectively inhibiting breast cancer cell proliferation [62]. Similarly, RSP-4–3-3 from rapeseed induces apoptosis and demonstrates antiproliferative action in liver cancer cells. P51, derived from plants, binds strongly to HER2 receptors, enhancing the precision of breast cancer therapies [63]. Bitter gourd peptide BG-4 promotes controlled cell death in colon and ovarian cancer [64], while olive seed and walnut residual peptideexhibit cytotoxic effects on prostate and breast cancers through apoptosis and cellular self-digestion [65]. Buckwheat-derived TBWSP31 regulates cell death in breast cancer [66], and the *Cycas revoluta* peptide induces apoptosis in colon carcinoma and epidermoid cancer cells [67]. Lunasin, a versatile peptide from soy and wheat, inhibits chromatin attachment and prevents malignant transformation in cancer cells triggered by oncogenes or chemical carcinogens [68]. Collectively, these peptides, as listed in Table 2, demonstrate diverse mechanisms such as apoptosis, cell cycle regulation, and receptor-specific targeting, highlighting their potential as natural therapeutic agents for various cancers.

**Table 2:** List of selected plant derived anti-cancer peptides

Peptide	Sequence	Target	Tumor	Mechanism	Source	References
NDF	MPACGSS	HCT-116, RKO and KM12L4 cell line	Human colon cancer	Suppression of cell proliferation and alteration of the expression of the proteins that control the cell cycle, including p53, p21, cyclin B1, BAD, cytC, c-casp3, survivin, and BIRC7	Common bean	[61]
CPe-III	RQSHFANAQP	MCF-7 and MDA-MB-231cell line	Breast cancer	Inhibits cell proliferation by elevating p53 levels	Chickpea	[62]
RSP-4–3-3	WTP (Trp-Thr-Pro)	HepG-2 cell line	Liver cancer	Apoptosis induction and antiproliferative action	Rapeseed	[63]
P51	CDTFPYLGWWNPNEYRY	HER2	Breast cancer	Indicates very strong binding to the HER2 receptors, improving the precision and effectiveness of the drug delivery	Plants	[76]
BG-4	_	HCT-116 and HT-29	Colon cancer	Controlled cell death	Bitter gourd	[64]
_	LLPSY	PC-3 and MDA-MB-468	Prostate and breast cancer	_	Olive seed	[65]
CTLEW	_	MCF-7	Breast cancer	Programmed cell death and Cellular self-digestion	Walnut residual	[77]
BG-4	_	A27801AP and COV318	Ovarian cancer	Cellular self-destruction	*Momordica charantia*	[78]
TBWSP31	_	Bcap37	Breast cancer	Regulated cell death	Buckwheat	[66]
Cycas revoluta peptide	AWKLFDDGV	HCT15 and Hep2	Colon carcinoma and human epidermoid cancer cells	Apoptosis	Palm fern seeds	[67]
Lunasin	SKWQHQQDSCRKQLQGVNLTPCEKHIMEKIQGRGDDDDDDDDD	Combat several cancer cell lines	Malignant mammalian cells that were brought on by viral oncogenes and chemical carcinogens	Attach to chromatin and inhibit transformation	Soy and wheat	[68]

### Microbial-derived peptides

Microbial-derived peptides in Table 3 exhibit a wide range of anti-cancer properties, targeting diverse pathways and tumor types. iPep624, derived from microbial sources, inhibits the transcription factor EN1, effectively targeting breast cancer [79]. Azurin, a bacterial protein, modulates p53 activity to deregulate proliferation and induce apoptosis across cancers like melanoma and breast carcinoma [83]. Similarly, Exotoxin A and Diphtheria toxin inhibit protein synthesis via ADP-ribosylation of elongation factor 2 (EF-2), showing efficacy against cancers such as pancreatic, melanoma, and glioblastoma [84]. Peptides like Pep27anal2 induce apoptosis and permeabilization in multiple cancers [99], while Entap promotes autophagic apoptosis in adenocarcinomas of the gastric, cervix, and mammary gland [86]. Actinomycin D [88] and Bleomycin [89], derived from Actinomyces and Streptomyces species, cause DNA damage through intercalation and free radical generation, targeting cancers such as sarcomas and ovarian carcinomas. Bestatin inhibits aminopeptidase N and functions as an adjuvant in metastasizing lymphoma models [93,94]. Novel peptides like Laterosporulin 10 and Pediocin K2a2-3 demonstrate dual apoptosis and necrosis induction, with potential in colon and breast cancer therapies [91]. Unique compounds like Apratoxin A from *Lyngbya majuscula* halt the cell cycle in G1 phase [97], while Beauvericin from Fusarium induces caspase-mediated apoptosis [98]. Collectively, these peptides highlight the therapeutic promise of microbial bioactive compounds in oncology.

**Table 3:** List of selected microbial source-derived anti-cancer peptides

Peptide	Sequence	Target	Tumor	Mechanism	Source	References
iPep624	KKKRKVTDSQQPLVWPAWVYCTRYSDRPS	Engrailed 1 (EN1)	Breast cancer	Inhibition of the transcription factor EN1	Microbial	[79]
MDP	L-Ala-D-isoGln	NOD protein	Solid tumour and liver	Stimulates the production of cytokines and interleukins to exhibit antitumor effects	Myco bacteria	[80]
Mixirins A–C	–	HCT-116	Human colon tumour	Blocked the growth of human colon tumor cell line (HCT-116)	*Bacillus* sp.	[81]
Heptapeptide	–	SK-MEL-28	Human melanoma	Cytotoxic activity	*Paenibacillus profundus*	[82]
Azurin	128 amino acids	p53	Breast cancer, melanoma, squamous carcinoma, reticulum cell sarcoma	Deregulation of proliferation and induction of caspase-dependent apoptosis	Microbial	[83]
Exotoxin A	638 amino acids	Elongation factor-2 (EF-2)	Pancreatic cancer, melanoma, head and neck squamous carcinoma, lung carcinoma, breast carcinoma, multiple myeloma	Inhibition of protein synthesis (ADP-ribosylation of cytoplasmic elongation factor 2)	Microbial	[84]
Pep27anal2	MWKWFHNVLSWWWLLADKRPARDYNRK	AML-2, HL-60, Jurkat, SNU-601 and MCF-7	Leukemia, gastric cancer, breast cancer	Cellular permeabilization, deregulation of proliferation and induction of caspase-independent apoptosis	Microbial	[30]
Urukthapelstatin A	–	(A549, DMS114), NCIH460, (OVCAR-3, OVCAR-4, OVCAR-5, OVCAR-8, SK-OV3), MCF-7, HCT-116	Human lung cancers, ovarian cancers, breast cancer, colon cancer	Dose-dependent growth inhibition of human lung tumor cells	*Mechercharimyces asporophorigenens* YM11-542	[85]
Entap	58-62 amino acids	AGS, HeLa, MDA-MB-231, 22Rv1 and HT-29	Gastric adenocarcinoma, uterine cervix adenocarcinoma, mammary gland adenocarcinoma, prostate carcinoma, colorectal adenocarcinoma	Deregulation of proliferation and induction of autophagous apoptosis	Microbial	[86]
Diphtheria toxin	538 amino acids	Elongation factor-2 (EF-2)	T cell lymphoma, glioblastoma, malignant brain tumors, adrenocortical carcinoma	Inhibition of protein synthesis (ADP-ribosylation of cytoplasmic elongation factor 2)	Microbial	[87]
Actinomycin D	1I3W	Several cell lines	Wilms cancer, Ewing sarcoma, neuroblastomas, trophoblastic tumours	Intercalation to DNA and the stabilization of cleavable complexes of topoisomerases I and II with DNA, photodynamic activity and free radical formation	*Actinomyces antibioticus*	[88]
Bleomycin	–	Several cell lines	Hodgkin’s disease, non-Hodgkin’s lymphoma, testicular carcinomas, ovarian cancer, malignant pleural effusion	Bleomycin (BLM) binds to DNA and Fe(II), generates hydroxyl radicals which induce DNA cleavage and cause Fe(II) to oxidize, leading to DNA damage	*Streptomyces verticillus*	[89]
Bovicin HC5	–	MCF-7, HepG2	Breast adenocarcinoma, liver hepatocellular carcinoma	Effective against spoilage bacteria and also inhibited growth of bacterial species	*Streptococcus bovis* HC5	[90]
Laterosporulin 10	–	HeLa, HEK293T, HT1080, H1299, MCF-7	Cervical cancer, embryonic kidney cancer, fibrosarcoma, lung carcinoma, breast cancer	At lower doses, this substance caused apoptosis of cancerous cells, while at higher doses it resulted in necrotic death of them	*Brevibacillus* sp. strain SKDU10	[91]
Pediocin K2a2-3	–	HT29	Colon adenocarcinoma	Growth inhibition of human colon adenocarcinoma cells (HT29)	*Pediococcus acidilactici* K2a2-3	[92]
Bestatin	2S, 3R-(3-amino-2-hydroxy-4-phenylbutanoyl)-l-leucine	Amino peptidase N	A number of tumour models, such as the model of metastasising ESb lymphoma	Functions both as an adjuvant and as an inhibitor of aminopeptidase N	Streptomyces	[93,94]
HVLSRAPR	Tr1–Tr4	MCF-7, HepG-2, SGC-7901 and HT-29	Breast, liver, gastric and colon	–	*Spirulina platensis*	[95]
CPAP	–	HepG2	Liver cancer	Apoptosis together with necrotic demise	*Chlorella pyrenoidosa*	[96]
Apratoxin A	–	HeLa and HT29	Cervical carcinoma and colon adenocarcinoma	Causing a cell cycle stop in the G1 phase	*Lyngbya majuscula*	[97]
Beauvericin	–	–	Human epidermoid carcinoma	Cytochrome C release and caspase-9/3 activation	*Fusarium* sp.	[98]

### Synthetic peptides

Synthetic peptides are a diverse class of therapeutic agents designed to target specific cancer-related pathways and structures, offering precision in treatment. M204C4 minimizes pancreatic cancer cell invasion by targeting matrix metalloproteinase-2 (MMP-2) [100]. Prostate-homing peptides deliver pro-apoptotic effects to prostate cancer tissues [101]. PNC-27 induces membrane lysis in breast cancer cells [102], while HRAP inhibits HER2-overexpressing cancer cell proliferation [103]. MAP-04-03 suppresses growth and cell migration in breast cancer, and Lyp-1 selectively binds tumor lymphatics and cells without affecting normal vessels [104]. D-K6L9 disrupts tumor development, and GTI enhances drug delivery specificity to prostate cancer cells by targeting prostate-specific membrane antigen (PSMA) [105]. Peptides like A5G81 promote cell attachment via integrins [106], while LinTT1 targets tumor endothelial cells through mitochondrial protein p32, aiding nanosystem-based therapies [107]. Pep42 targets glucose-regulated protein 78 (GRP78) in melanoma cells offering specificity, while Bld-1 binds selectively to bladder tumor tissues, enabling targeted diagnostics and treatment [108]. ATN-161 and Cilengitide inhibit integrin interactions, reducing tumor cell adhesion and angiogenesis in prostate and various cancers [109]. Anti-angiogenic peptides such as CGNSNPKSC suppress blood vessel growth in gastric cancer [110], while cyclic nonapeptides target aminopeptidase-P to localize conjugated drugs in breast cancer tissue [111]. The binding peptide TLTYTWS reduces angiogenesis in lung cancer models, and TM peptide inhibits tumor growth, angiogenesis, and migration in breast cancer by targeting neuropilin-1 (NRP1) receptors [112]. Together, these peptides exemplify the versatility of synthetic molecules in addressing cancer complexities through tailored mechanisms as listed in Table 4.

**Table 4:** List of selected synthetic anti-cancer peptides

Peptide	Sequence	Target	Tumor	Mechanism	Source	References
M204C4	HWWQWPSSLQLRGGGS	MMP-2	Pancreatic cancer	Minimize the invasion of tumor cells by MMP-2	Synthetic	[100]
PNC-27	PPLSQETFSDLWLLKKWKMRNQFWVKVQRQ	MCF-7	Breast cancer	Induce membrane lysis	Synthetic	[102]
HRAP	Ac-PHAHF-NH2	HER2	Breast cancer	Inhibits the proliferation of HER2-overexpressed cancer cells	Synthetic	[103]
Lyp-1	CGNKRTRGC	Mitochondrial protein p32	Breast cancer	Binds to and accumulates in tumor lymphatics and tumor cells, but not normal blood vessels	Synthetic	[104]
D-K6L9	LKLLKKLLKKLLKLL	Cytoplasmic membrane	Primary/metastatic tumors	Inhibiting the development of human tumors	Synthetic	[105]
GTI	GTIQPYPFSWGY	Prostate-specific membrane antigen (PSMA)	Prostate cancer	Improves the specificity and efficiency of drug delivery to prostate cancer cells	Synthetic	[126]
LinTT1	AKRGARSTA	Mitochondrial protein p32	Breast cancer	Binds to p32/gC1qR on tumor endothelial cells, helping the nano system target tumor vessels	Synthetic	[107]
Pep42	CTVALPGGYVRVC	GRP78	Melanoma cells	Specifically targets GRP78-expressing cancer cells,	Synthetic	[127]
Bld-1	CSNRDARRC	Bladder tumor cells	Bladder cancer	Selectively binds to bladder tumor cells and tissues, enabling targeted therapy and diagnostic detection of bladder cancer	Synthetic	[108]
ATN-161	PHSCN	Integrin α5β1	Human prostate carcinoma	Stops fibronectin from interacting with the α5β1 site	Synthetic	[109]
Cilengitide	RGDFV	Integrin αVβ3	Melanoma, prostate cancer	High-affinity αVβ3 inhibitor based on cyclic RGD	Synthetic	[128]
Cyclic peptide	CPGPEGAGC	Aminopeptidase-P	Human breast cancer	Apply a conjugated medication to the tumor’s tissue	Synthetic	[129]
Binding peptide	TLTYTWS	Collagen IV	Lewis lung cancer in mice	Diminish angiogenesis and endothelial differentiation	Synthetic	[130]
TM peptide	ILITIIAMSALGVLLGAVCG VVLYRKR	Neuropilin-1 receptor	Human breast cancer	Reduced tumor growth, angiogenesis, cancer migration	Synthetic	[112]

### Stapled peptides as anti-cancer agent

Stapled peptides are designed by introducing chemical cross-links, or “staples,” to stabilize their α-helical structures, which are crucial for binding to target proteins. This stabilization enhances their resistance to proteolytic degradation and improves cell membrane permeability, making them effective in modulating intracellular protein-protein interactions (PPIs) [113]. The synthesis of stapled peptides commonly involves ring-closing metathesis (RCM) to introduce hydrocarbon staples. This method incorporates non-natural amino acids with olefinic side chains into the peptide sequence, followed by a catalytic reaction to form the staple. Alternative stapling techniques, such as lactam bridges and disulfide bonds, have also been explored to enhance peptide stability and functionality [114]. They have shown significant promise in cancer therapy. For instance, a stapled peptide targeting the MDM2-p53 interaction demonstrated potent antitumor activity by reactivating p53 function in cancer cells [115]. Additionally, stapled peptides designed to inhibit the interaction between the transcription factor STAT3 and its co-activators have been developed, leading to the suppression of tumor growth in preclinical models [116]. The primary advantages of stapled peptides include enhanced stability, improved cell permeability, and the ability to target PPIs with high specificity. However, challenges such as optimizing their pharmacokinetic properties and ensuring efficient delivery to target tissues remain [117]. This class of peptides represent promising anticancer agents, offering the ability to modulate challenging PPIs with enhanced stability and specificity.

## Modes of Action

### Cell membrane disruption

**Figure 1:**
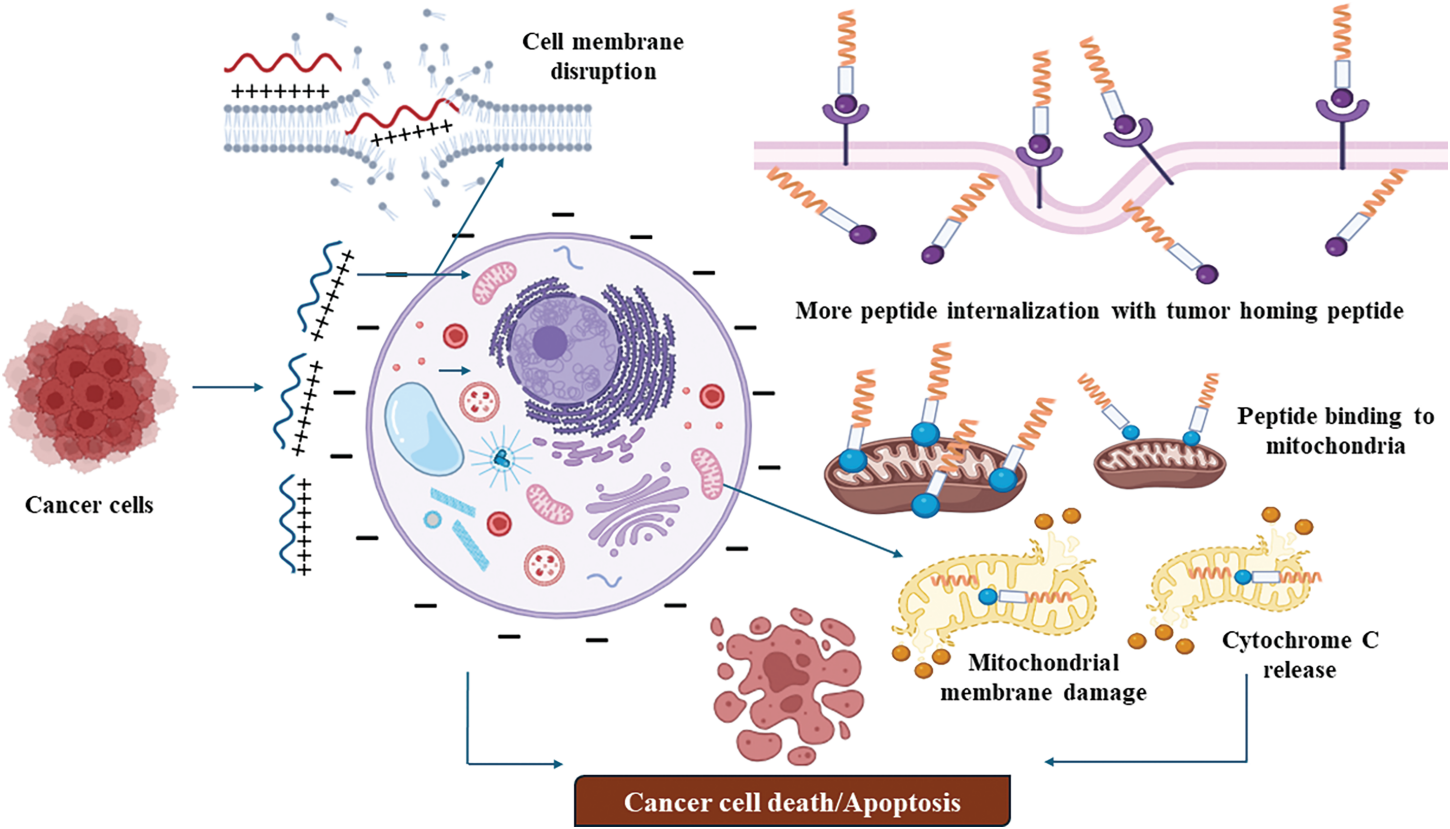
Membrane disrupting mechanism of tumor targeting peptides (created on Biorender, accessible at https://biorender.com/).

Certain tumor-suppressive peptides, such as LL-37 [118] and melittin [119], exhibit amphipathic and cationic properties that enable them to interact with the negatively charged membranes of cancer cells. LL-37, a human cathelicidin peptide, and BMAP-28, a bovine-derived peptide, destabilize these membranes due to their electrostatic interactions, ultimately leading to cell lysis. Cancer cells, with their altered lipid composition and higher negative charge, are more vulnerable to these peptides compared to normal cells (Fig. 1). This selective mechanism allows these peptides to target cancer cells effectively while sparing healthy cells, demonstrating their potential for anticancer therapy.

#### Enhanced internalization through tumor-homing peptides

**Figure 2:**
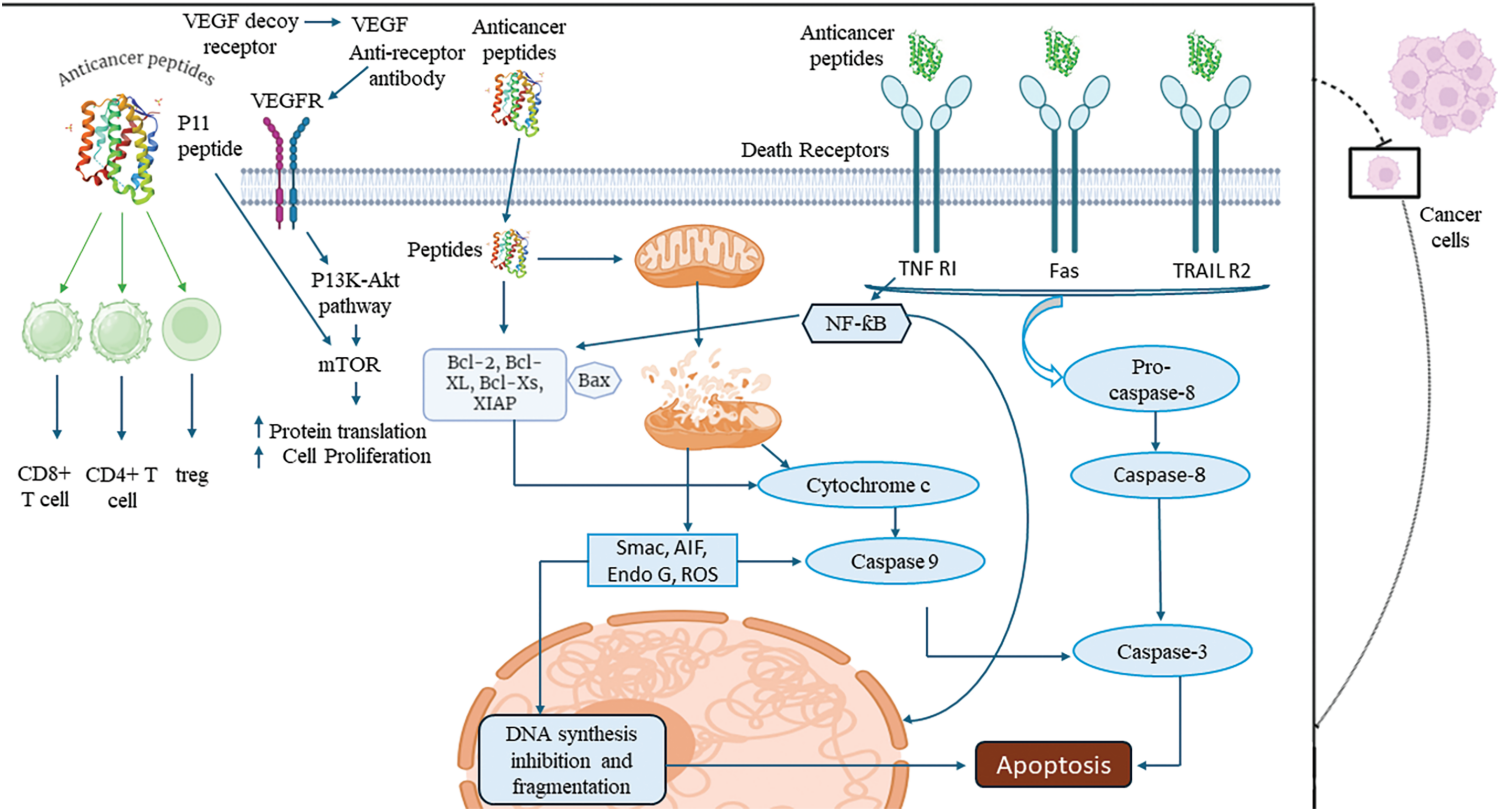
An overview of apoptotic mechanisms of tumor targeting peptides (created on Biorender, accessible at https://biorender.com/). VEGFR: vascular endothelial growth factor receptor, TNF R1: tumor necrosis factor receptor 1, TRAIL R2: tumor necrosis factor-related apoptosis-inducing ligand receptor 2, mTOR: mechanistic target of rapamycin, Bcl-2: B-cell lymphoma 2, Bcl-xL: B-cell lymphoma-extra large, Caspase-8: cysteine-aspartic protease 8, CD8+ T cell: cluster of differentiation 8 positive T cell, CD4+ T cell: cluster of differentiation 4 positive T cell, Treg: regulatory T cell, Smac: second mitochondria-derived activator of caspases, AIF: apoptosis-inducing factor, Endo G: endonuclease G, ROS: reactive oxygen species, Caspase-3: cysteine-aspartic protease 3, DNA: deoxyribonucleic acid, XIAP: X-linked inhibitor of apoptosis protein.

Tumor-homing peptides enhance the targeted delivery and therapeutic efficacy of anticancer peptides by recognizing specific markers or receptors overexpressed on tumor cells or within the tumor microenvironment. These peptides bind selectively to these markers, ensuring precise delivery and deep penetration into tumor tissues, overcoming barriers such as the extracellular matrix. For example, RGD peptides (arginine-glycine-aspartic acid) target integrins, such as αvβ3 and αvβ5, which are commonly overexpressed on the surface of tumor cells and tumor vasculature [120]. Similarly, iRGD peptides, which contain a tumor-penetrating motif, bind to integrins and subsequently interact with neuropilin-1 receptors, facilitating deeper tissue penetration and intracellular delivery of anticancer agents [121]. Another example is F3 peptide, which binds to nucleolin, a marker overexpressed on tumor cells and angiogenic blood vessels, enhancing the uptake of therapeutic agents specifically into cancerous tissues [122] (Fig. 2). These mechanisms ensure selective targeting and efficient therapeutic action with minimal impact on healthy cells.

#### Mitochondrial targeting

Some peptides specifically target mitochondrial proteins within cancer cells, disrupting mitochondrial membrane integrity and triggering apoptosis. For instance, p53-derived peptides mimic the tumor-suppressor protein p53 and bind to anti-apoptotic proteins like Bcl-2 and Bcl-xL on the mitochondrial membrane, promoting cytochrome C release and intrinsic apoptotic pathway activation [123]. Similarly, KLAKLAK-2, an antimicrobial peptide, targets the mitochondrial membrane directly, compromising its potential and inducing cytochrome C release [124]. Another example is the BID BH3 peptide, which interacts with pro-apoptotic proteins like Bax or Bak, facilitating mitochondrial outer membrane permeabilization (MOMP) and initiating apoptosis [125] (Fig. 2). This strategy has been explored for treating various cancers, including breast cancer, lung cancer, pancreatic cancer, and melanoma, where mitochondrial dysfunction and overexpression of anti-apoptotic proteins are common, ensuring selective and effective cancer cell death.

#### Apoptosis induction via cytochrome C release

Peptides that disrupt mitochondrial integrity initiate apoptosis by releasing cytochrome C into the cytoplasm, where it binds to apoptotic protease activating factor-1 (Apaf-1) to form the apoptosome, activating caspase-9. This activation triggers caspase-3, leading to DNA fragmentation, chromatin condensation, and programmed cell death. This mechanism selectively eliminates cancer cells while sparing healthy tissues. For example, Bcl-2-converting peptide (Bcl-2CP) binds to anti-apoptotic Bcl-2 family proteins, neutralizing their function and promoting mitochondrial membrane permeabilization [131]. MTP-131 (also known as Bendavia) targets mitochondrial membranes, causing disruption and cytochrome C release, effectively inducing apoptosis [132]. Another example is MAC (Mitochondrial apoptosis-induced channel), which disrupts the mitochondrial membrane potential and triggers cytochrome C release. This strategy has demonstrated potential in treating cancers such as prostate cancer, breast cancer, gastric cancer, and multiple myeloma, where mitochondrial resistance mechanisms are frequently observed [133] (Fig. 2). This strategy has demonstrated potential in treating cancers such as prostate cancer, breast cancer, gastric cancer, and multiple myeloma, where mitochondrial resistance mechanisms are frequently observed.

#### VEGF pathway inhibition

Certain peptides inhibit the vascular endothelial growth factor (VEGF) signaling pathway by specifically binding to the VEGF receptor (VEGFR), preventing its activation. This inhibition blocks the downstream PI3K-Akt-mTOR signaling cascade, a critical pathway for tumor angiogenesis, growth, and survival [134]. By disrupting angiogenesis, these peptides deprive tumors of essential nutrients and oxygen, effectively curbing tumor growth and reducing metastatic potential. For example, SP5.2, a peptide antagonist of VEGFR-2, binds to the receptor and inhibits VEGF-mediated angiogenic signaling [135]. Similarly, A7R peptide targets VEGFR-2 and neuropilin-1, impairing endothelial cell proliferation and migration necessary for blood vessel formation [136]. CBO-P11 peptide binds directly to VEGFR-1, suppressing VEGF signaling and reducing vascular permeability [137] (Fig. 2). This strategy has shown promise in targeting cancers such as colorectal cancer, breast cancer, non-small cell lung cancer, and glioblastoma, where VEGF-driven angiogenesis plays a pivotal role in tumor progression and metastasis.

#### Death receptor activation

Peptides targeting death receptors such as TNF-RI, Fas, and TRAIL R2 on the surface of cancer cells activate the extrinsic apoptotic pathway. These peptides bind to death receptors, inducing receptor trimerization and recruiting adaptor proteins like FADD (Fas-associated death domain). This process facilitates the conversion of procaspase-8 into active caspase-8, which subsequently activates caspase-3. Activated caspase-3 executes apoptosis by cleaving essential structural and regulatory proteins, ultimately leading to cancer cell death [138]. This pathway effectively bypasses some resistance mechanisms that cancer cells employ to evade intrinsic apoptosis. For example, Apo-2L/TRAIL-mimetic peptides mimic the TRAIL ligand and bind specifically to TRAIL R2 (DR5), inducing selective apoptosis in cancer cells without affecting normal cells [139]. Similarly, FasL-derived peptides activate the Fas receptor, promoting apoptotic signaling. Another example is TANDEM peptide, which targets TNF-RI, triggering apoptotic cascades in cancer cells [140] (Fig. 2). This strategy is particularly effective in treating cancers such as colorectal cancer, pancreatic cancer, non-small cell lung cancer, and prostate cancer, where death receptor expression is upregulated, making these tumors susceptible to extrinsic apoptotic activation.

#### Immune modulation

Some tumor-suppressive peptides enhance the activity of immune effector cells, such as CD8+ cytotoxic T cells and D4+ helper T cells, while simultaneously suppressing immunosuppressive regulatory T cells (Tregs). By modulating the tumor microenvironment, these peptides reprogram the immune response, boosting the body’s natural ability to recognize and eliminate cancer cells. This dual action strengthens antitumor immunity and contributes to long-term tumor suppression by promoting immune surveillance. For example, cytotoxic T lymphocyte-associated antigen 4 (CTLA-4)-blocking peptides, such as specific peptide fragments derived from ipilimumab, enhance CD8+ T cell activation by inhibiting CTLA-4-mediated immune suppression [141]. MAGE-A3-derived peptides stimulate CD4+ T helper cells, enhancing cytokine production and promoting cytotoxic T cell responses [142]. Additionally, forkhead box P3 (FOXP3)-targeting peptides reduce Treg activity, reversing the immunosuppressive environment that allows tumor cells to evade immune detection [143] (Fig. 2). This strategy is effective in cancers such as melanoma, non-small cell lung cancer, renal cell carcinoma, and head and neck squamous cell carcinoma, where immune evasion is a significant barrier to effective treatment. By enhancing effector T cell activity and suppressing Tregs, these peptides provide a potent mechanism for boosting the immune system’s antitumor response.

#### DNA synthesis inhibition and fragmentation

Peptides targeting cancer cell nuclei interfere with DNA synthesis, a critical process for tumor cell proliferation. By localizing to the nucleus, these peptides induce DNA fragmentation, disrupt the replication machinery, and halt cancer cell growth [144]. This mechanism complements other apoptotic pathways, ensuring that cancer cells are eliminated through multiple avenues, thereby enhancing therapeutic efficacy. NLS (nuclear localization signal)-tagged pro-apoptotic peptides specifically target the nucleus to induce DNA fragmentation and inhibit replication enzymes like DNA polymerase [145]. BuGZ peptide derivatives disrupt the assembly of the spindle checkpoint complex, leading to DNA replication stress and cell death [146]. Intranuclear lytic peptides (e.g., LTX-315) penetrate the nucleus, disrupt chromatin structure, and induce DNA damage, leading to apoptosis [147]. Another example is TAT-conjugated DNA-intercalating peptides, which penetrate the nucleus and bind to specific sequences, preventing DNA polymerase activity [148] (Fig. 2). This strategy is effective in cancers such as breast cancer, glioblastoma, leukemia, and cervical cancer, where rapid DNA synthesis drives tumor progression. By halting replication and inducing DNA damage, nuclear-targeting peptides provide a targeted and potent approach to cancer therapy either as standalone treatments or in combination or in combination with other therapeutic agents.

### Microenvironment barriers to peptide therapy

The tumor microenvironment (TME) significantly influences the efficacy of peptide-based cancer therapies which can affect both the targeting capabilities and pharmacokinetics of therapeutic peptides. Tumor hypoxia, a condition of reduced oxygen levels, is a hallmark of the TME. Hypoxic conditions can lead to the stabilization of hypoxia-inducible factors, which in turn modulate the expression of various genes involved in angiogenesis, metabolism, and cell survival. These changes can alter the behavior of cancer cells and the surrounding stroma, potentially impacting the distribution and effectiveness of peptide-based therapies [149]. The TME is often characterized by an acidic extracellular pH, resulting from increased glycolysis and subsequent lactate production by cancer cells. This acidic environment can affect the stability and activity of therapeutic peptides. However, it also presents an opportunity for designing pH-responsive delivery systems. For example, pH (low) insertion peptides can exploit the acidic conditions to selectively target tumor cells, enhancing the delivery and efficacy of peptide-based treatments [150]. The extracellular matrix in tumors is often denser and more rigid than in normal tissues, due to increased deposition and cross-linking of matrix components. This dense matrix can act as a physical barrier, hindering the penetration and distribution of therapeutic peptides within the tumor. Additionally, remodeling of dense matrix can influence tumor progression and metastasis, further complicating treatment strategies [151]. All these conditions can pose significant challenges to peptide-based cancer therapies. However, by designing peptides and delivery systems that specifically target these features, it is possible to enhance therapeutic efficacy and overcome some of the barriers.

### Advanced peptide delivery in cancer therapy

Advanced peptide drug delivery systems, including nanoparticles, liposomes, and polymers, have been developed to enhance the targeting, stability, and therapeutic efficacy of peptides in cancer treatment.

#### Nanotechnology

Nanoparticles, typically ranging from 10 to 100 nm, offer several advantages for peptide delivery [152] including enhanced stability by encapsulating peptides within nanoparticles protecting them from enzymatic degradation and premature clearance, thereby prolonging their circulation time. Another strategy is surface modifications with targeting ligands, which enable nanoparticles to bind specifically to tumor cells, improving the precision of drug delivery. For instance, conjugating nanoparticles with peptides like RGD can facilitate the targeting of integrin receptors overexpressed on tumor cells [153]. Nanoparticles can be engineered to release their payload in response to specific stimuli, such as pH changes or enzymatic activity, ensuring that peptides are released at the tumor site [154]. One example is the targeted delivery of anticancer peptides like melittin by using poly lectic-co-glycolic acid nanoparticles, which protect them from enzymatic degradation and enable controlled release in tumors [155].

#### Liposomal strategy

Liposomes are spherical vesicles composed of lipid bilayers that can encapsulate peptides and are well-tolerated by the body, reducing the risk of adverse reactions [152]. Liposomes can improve the penetration of peptides into tumor tissues by facilitating cellular uptake and further functionalizing liposomes with targeting peptides or antibodies can enhance specificity to tumor cells, thereby increasing therapeutic efficacy [156]. Doxil, a liposome-based system encapsulating doxorubicin, enhances drug biocompatibility, reduces toxicity, and improves tumor penetration. Similar liposomal approaches protect therapeutic peptides and enable targeted cancer cell delivery [157].

#### Polymeric strategy

In recent years, natural and synthetic polymer-based delivery systems have emerged to enhance drug targeting, prolong circulation, and enable controlled release. These biodegradable and biocompatible polymers improve drug stability and reduce side effects through mechanisms like adsorption, conjugation, and internal loading [158]. Polymers can be synthesized with various functional groups, allowing for customization of drug delivery systems. Many are biodegradable, minimizing long-term toxicity concerns. Polymers can be designed to respond to specific stimuli, such as pH or temperature, enabling controlled release of peptides at the tumor site [159]. Polymeric micelles are another effective system for peptide drug delivery. For instance, pluronic F-127, a triblock copolymer, has been used to form micelles for encapsulating peptides like *melittin* for targeted cancer therapy. These micelles are capable of responding to external stimuli such as pH changes in the tumor microenvironment, leading to controlled release of the peptide specifically at the tumor site [160].

## Applications in Cancer Therapy: FDA-Approved Anti-Cancer Peptides

### Standalone peptide therapies

This section provides a comprehensive overview of FDA-approved ACPs, highlighting their manufacturers, target tissues, and mechanisms of action. The use of peptides underscore the potential of targeted cancer therapies in modern oncology, offering advantages such as specificity, reduced off-target effects, and improved therapeutic outcomes. Carfilzomib (Kyprolis) produced by Amgen, Carfilzomib is a proteasome inhibitor targeting malignant plasma cells, making it a key treatment for multiple myeloma. By disrupting protein degradation pathways essential for tumor survival, it induces cancer cell apoptosis. Its success underlines the potential of proteasome inhibitors in hematologic malignancies [161]. SomaKit TOC manufactured by SomaKit TOC, this drug is specifically designed for gastroenteropancreatic neuroendocrine tumors. Its precise targeting of somatostatin receptors enhances diagnostic and therapeutic strategies for this rare cancer type, showcasing the utility of receptor-targeted peptides [162]. Venetoclax (Venclexta): AbbVie developed Venetoclax as a selective inhibitor of BCL-2, a protein that prevents apoptosis in cancer cells. By targeting this anti-apoptotic pathway, Venetoclax has proven effective in treating hematologic cancers like chronic lymphocytic leukemia, reflecting the promise of apoptosis-restoring therapies [163]. Osimertinib (Tagrisso): Produced by AstraZeneca, Osimertinib is a third-generation tyrosine kinase inhibitor (TKI) targeting EGFR mutations, a frequent driver of non-small cell lung cancer. It offers benefits over first- and second-generation TKIs, such as improved blood-brain barrier penetration and reduced resistance development, making it a cornerstone in lung cancer therapy [164]. Doxil, a liposomal formulation of doxorubicin by Sequus Pharmaceuticals, Doxil is used for breast cancer, ovarian cancer, and other solid tumors. Encapsulation within liposomes minimizes systemic toxicity and cardiac side effects, exemplifying how nanotechnology enhances peptide-based chemotherapies’ safety profiles [165]. Aprepitant (Emend®): Developed by merck sharp and Dohme BV, Aprepitant is primarily used for its anti-emetic properties in cancer treatment. While it targets neurokinin-1 receptors, its role in lung, neuroendocrine, and gastric cancers demonstrates the potential of dual-function drugs that address cancer symptoms and progression [166]. Cisplatin and Cisplatinum: Manufactured by Cadila Pharmaceuticals, these platinum-based compounds are staples in chemotherapy for bladder, head and neck, lung, ovarian, and testicular cancers. Despite their broad-spectrum activity, their high toxicity levels highlight the ongoing need for safer alternatives or combination therapies to minimize side effects [167].

Many of these agents work by disrupting critical pathways in cancer cells, such as proteasome activity (Carfilzomib) or apoptotic resistance (Venetoclax). Others, like SomaKit TOC, focus on receptor-specific targeting, illustrating how tailored mechanisms enhance therapeutic efficacy. Although these peptides are FDA-approved, challenges remain, including resistance development (e.g., Osimertinib in lung cancer) and systemic toxicities (e.g., Cisplatin). Advanced formulations like Doxil showcase the potential of nanotechnology to address such limitations. The success of these peptides points to the need for continued research into combination therapies, next-generation formulations, and targeted delivery systems. Expanding their application to underexplored cancers could further enhance their impact on global oncology.

### Peptide-based in fusion cancer therapy

Advances in anticancer therapies have introduced novel treatments that leverage antibody-drug conjugates (ADCs), radioligands, and peptide-based agents to target specific cancer pathways with improved efficacy and reduced off-target effects. These innovative approaches integrate conjugated peptides and proteins to enhance tumor specificity and therapeutic potential, marking a significant shift in precision oncology. Some notable examples of such therapies, including their conjugated peptide components and clinical outcomes are discussed in this section. Sacituzumab govitecan, an antibody-drug conjugate targeting Trophoblast cell surface antigen 2 (Trop-2), demonstrated remarkable efficacy in treating metastatic triple-negative breast cancer. A phase 3 trial revealed significant improvements in progression-free survival and overall survival compared to standard chemotherapy [168]. Lutetium-177–Dotatate has emerged as a potent treatment for advanced midgut neuroendocrine tumors when a somatostatin analog peptide was conjugated with Leutetium-177. Clinical trials showed markedly extended progression-free survival and higher response rates compared to high-dose octreotide long acting release (LAR). While mild myelosuppression was noted, the absence of renal toxicity emphasized its safety and therapeutic potential [169]. Radioligand therapy with 177Lu-PSMA-617 (Lutetium-177 joined with a prostate-specific membrane antigen (PSMA)-targeting peptide), combined with standard care, significantly improved progression-free survival and overall survival in patients with advanced PSMA-positive metastatic castration-resistant prostate cancer. Though associated with grade 3 or higher adverse events, such as hematologic toxicities, the treatment did not affect the quality of life, solidifying its role in this patient population [169]. Denileukin diftitox, a fusion protein conjugate, combines diphtheria toxin fragments with interleukin-2 and targeting IL-2 receptors, exhibited efficacy in patients with persistent or recurrent cutaneous T-cell lymphoma. A phase 3 study reported a 30% overall response rate, with durable responses. The treatment was associated with flu-like symptoms, vascular leak syndrome, and transient hepatic enzyme elevations but was considered tolerable [170]. Rovalpituzumab tesirine, a DLL3-targeting antibody-drug conjugate, showed encouraging antitumor activity in DLL3-expressing small-cell lung cancer during phase 1 trials. An 18% objective response rate was observed in patients treated at effective doses, with manageable toxicity profiles, supporting its further exploration [171]. Ramucirumab, in combination with FOLFIRI, significantly improved overall survival in metastatic colorectal cancer patients as a second-line treatment. The RAISE trial reported consistent benefits across subgroups with a manageable safety profile, validating its role in this therapeutic setting [172].Ongoing research in fusion protein technology is set to transform the landscape of cancer treatment, offering more effective and personalized therapeutic options.

## Computational Tools for Protein Modeling and Analyzing Their Therapeutic Potential

### Protein modeling software and online tools

Protein modeling tools and online platforms play a vital role in understanding the structure-function relationship of biomolecules. AlphaFold 2 [173], integrated into platforms like The Human Protein Atlas, has transformed structural biology by employing artificial intelligence to predict protein 3D structures with near-experimental accuracy. It enables researchers to investigate protein mechanisms, interactions, and pathways efficiently. SWISS-MODEL [174], an automated homology modeling server, identifies structural templates from databases and builds reliable protein models based on sequence alignment [175]. This tool simplifies the modeling process for proteins with known homologous structures. PyMOL [176], widely used for visualizing biomolecules, also includes plugins for specialized tasks such as antigenicity prediction, aiding in vaccine and immunotherapy design. MOE [177] (Molecular Operating Environment) integrates molecular modeling and cheminformatics, providing capabilities for calculating molecular weight, protein-ligand docking, and drug design. Another notable tool, trRosetta [178], uses deep learning to predict inter-residue orientations and distances, facilitating *de novo* protein structure prediction. Together, these tools provide a comprehensive suite for studying, visualizing, and interpreting protein structures, making them indispensable for structural biology, drug discovery, and bioinformatics research.

### Protein structure validation tools

Protein structure validation ensures the accuracy and reliability of modeled or experimentally derived 3D structures. PROCHECK [179] is a gold-standard tool that evaluates stereochemical parameters of protein models, providing detailed Ramachandran plots to highlight deviations in torsion angles [180]. These insights are crucial for identifying potential modeling errors. MolProbity [181], another powerful validation tool, offers a wide range of structural checks, including the detection of steric clashes, assessment of rotamer quality, and evaluation of backbone dihedral angles. Its ability to provide atomic-level validation makes it an essential tool for refining protein structures. Additional tools, such as QMEAN [182], provide a quantitative estimate of model quality by comparing the predicted structure to experimental data, while Verify3D evaluates the compatibility of the 3D structure with its amino acid sequence, ensuring the model’s reliability for functional studies [183,184]. These validation methods are essential steps before proceeding with downstream applications such as docking, molecular dynamics simulations, or experimental studies, as they ensure that the protein structure accurately reflects its biological reality.

### Docking software

Protein docking tools are essential for predicting and analyzing peptide-receptor interactions, offering a range of sophisticated algorithms for accurate binding predictions. ClusPro, a widely used docking tool, applies clustering algorithms to identify energetically favorable protein-protein complexes, making it ideal for studying large macromolecular assemblies [185] HADDOCK [186,187] (High Ambiguity Driven DOCKing) goes beyond rigid-body docking by incorporating experimental data such as nuclear magnetic resonance (NMR) and mutagenesis studies, enabling flexible docking of proteins, DNA, and small molecules. Z-Dock [188] specializes in rigid-body docking for protein-protein interactions and uses a scoring system based on shape complementarity and electrostatics to predict optimal orientations. MOE (Molecular operating environment) offers comprehensive docking capabilities, including flexible ligand handling, advanced scoring functions, and customizable workflows, making it highly versatile for drug discovery projects [189]. Geometry-based docking tools like PatchDock [190] detect surface complementarity between interacting molecules, providing a quick and efficient method for docking large complexes. Chimera [191], while primarily a visualization tool, integrates docking capabilities through plugins like AutoDock Vina, allowing researchers to study binding poses and interaction patterns in detail [192]. These tools cater to diverse docking requirements, facilitating the exploration of biomolecular interactions in structural biology, biochemistry, and drug discovery.

### Simulation tools

Molecular dynamics (MD) simulations offer a dynamic perspective on biomolecular behavior, revealing insights into conformational flexibility, binding mechanisms, and stability under physiological conditions. GROMACS [193], renowned for its high performance, is extensively used for simulating proteins, lipids, and nucleic acids. Its efficient algorithms and advanced trajectory analysis tools make it a preferred choice for large-scale MD studies. NAMD [194] excels in handling complex systems, leveraging parallel computing to simulate large biomolecular assemblies, such as solvated protein-ligand complexes, with high computational efficiency. Desmond, part of the Schrödinger suite, provides an intuitive user interface combined with high-accuracy molecular dynamics simulations, making it suitable for studying detailed protein-ligand dynamics and exploring conformational changes. AMBER [195] specializes in using precise force fields to simulate biomolecular interactions accurately and supports advanced parameterization for custom molecules. These tools are often complemented by enhanced sampling techniques, such as metadynamics and free energy calculations, to explore rare events and binding affinities [196]. Molecular simulation tools are invaluable for understanding the dynamic nature of biomolecular systems, predicting their behavior under different conditions, and validating hypotheses generated from static models or docking studies.

### Key contributions of bioinformatics in anti-cancer drug design of fusion proteins

A vast amount of published literature exists on this topic, and while it is not possible to cover all of it due to the scope of this review, a selection of key studies will be highlighted to emphasize their importance in the field. Rehman et al. (2024) employed *in silico* methods to develop an Azurin-BR2 chimeric protein, showcasing high binding affinity to p53 and potential apoptotic induction in cancer cells [197]. Similarly, Khalid et al. (2024) designed a Leptulipin-p28 fusion protein, targeting VEGFR and Cadherin receptors, which demonstrated stability and interaction efficacy in molecular dynamics simulations [198]. In another study, Rehman et al. (2023) reported the *in-silico* design of a melittin-IL-24 fusion protein, validated through structure quality indices and simulation studies, indicating promising therapeutic potential [199]. Azurin’s anticancer efficacy was further emphasized by Aslam et al. (2024), combining *in silico* predictions and *in vitro* validations to confirm its cytotoxicity against breast cancer cells [200]. Qureshi et al. (2024) explored an IL-24-P20 fusion protein, revealing exceptional stability and receptor interaction capabilities [201]. Fatima et al. (2024) computationally engineered an IL-15-NGR peptide, demonstrating targeted cancer cell homing and stability through advanced docking and simulation techniques [202]. Moreover, Rehman et al. (2024) introduced an IL-24-NBD peptide fusion, highlighting successful receptor interaction and apoptotic potential [203]. Earlier studies, such as Moghadam et al. (2019), focused on DT390-STxB constructs, presenting optimized codon usage and structural stability for efficient translation and anti-tumor activity [204]. Goleij et al. (2019) demonstrated the robust interaction of Herceptin-based fusion proteins with HER2 receptors, proposing their candidacy as novel anticancer agents [205]. *In silico* analysis offers a safe and efficient approach to studying infectious organisms, minimizing the risk of exposure to hazardous pathogens. Collectively, these studies validate the critical role of computational approaches in designing and optimizing fusion proteins for cancer therapy, paving the way for targeted and effective treatments.

## Conclusion and Future Perspective

Future research in peptide-based cancer therapy should focus on optimizing peptide design, enhancing therapeutic efficacy, and addressing current limitations to improve clinical outcomes. Peptide modifications such as cyclization, pegylation, and the incorporation of non-natural amino acids can enhance stability and prolong circulation time. Advanced delivery strategies, including nanoparticles, liposomes, and polymeric micelles, can improve bioavailability, while stimuli-responsive systems can enable controlled release at tumor sites. Combination therapies with immune checkpoint inhibitors or chemotherapy may further enhance treatment efficacy. Addressing challenges such as drug resistance, off-target effects, and immunogenicity is crucial for successful clinical translation. Additionally, integrating AI-driven computational modeling and personalized medicine approaches can help tailor peptide therapies to individual patient profiles. By overcoming these challenges, peptide-based therapies have the potential to become a key component of future cancer treatment strategies.

## Data Availability

The data and materials employed in this study can be obtained from the corresponding author upon request.

## Abbreviations

BCR: Breakpoint cluster region
ABL: Abelson murine leukemia
CML: Chronic myeloid leukemia
ACPs: Anticancer peptides
EN1: Engrailed 1
EF-2: Elongation factor-2
MOMP: Mitochondrial outer membrane permeabilization
VEGF: Vascular endothelial growth factor
FADD: Fas-associated death domain
Tregs: Regulatory T cells
NLS: Nuclear localization signal
TKI: Tyrosine kinase inhibitor
HER2: Human epidermal growth factor receptor 2
NF-κB: Nuclear Factor kappa-light-chain-enhancer of activated B cells
MMP-2: Matrix Metalloproteinase-2
PNA: Peptide nucleic acid
Apaf-1: Apoptotic protease activating factor-1
GRP-78: Glucose-Regulated Protein 78
NRP-1: Neuropilin-1
MAC: Mitochondrial apoptosis-induced channel
CTLA-4: Cytotoxic T Lymphocyte-Associated Antigen 4
FOXP3: Forkhead Box P3
Trop-2: Trophoblast cell surface antigen 2
LAR: Long acting release
NMR: Nuclear magnetic resonance
HTS: High throughput screening
ADCs: Antibody-drug conjugates
PPIs: Protein-protein interactions
Fab: Antigen binding fragment
ScFV: Single chain variable fragment
FACS: Fluorescence-activated cell sorting
RCM: Ring-closing metathesis
TME: Tumor microenvironment
PSMA: Prostate-specific membrane antigen
MOE: Molecular Operating Environment
HADDOCK: High Ambiguity Driven DOCKing
MD: Molecular dynamics
XIAP: X-linked inhibitor of apoptosis protein
